# Immunosuppression of spleen in mice treated with erythropoietin: transcriptomic and immunological analysis

**DOI:** 10.3389/fimmu.2025.1560589

**Published:** 2025-03-21

**Authors:** Xinyi Lyu, Jiahao Shi, Qi Liu, Mingjun Jiang, Xilian Liu, Yulan Li, Shuqin Ding, Xianpeng Dai

**Affiliations:** ^1^ The Second Affiliated Hospital, Department of Vascular Surgery, Hengyang Medical School, University of South China, Hengyang, China; ^2^ The Second Affiliated Hospital, Department of Endocrinology and Metabolism, Hengyang Medical School, University of South China, Hengyang, China; ^3^ Clinical Laboratory, The First Affiliated Hospital of Bengbu Medical University, Bengbu, Anhui, China

**Keywords:** erythropoietin, splenomegaly, RNA sequencing, immunosuppression, proliferation

## Abstract

**Background and aim:**

Long term high-dose erythropoietin (EPO) had been reported inducing the formation of abdominal aortic aneurysm (AAA) in mice. When using this model, we found that EPO treated mice showed significant splenomegaly. This is an interesting phenomenon, and its mechanism has not been reported. Therefore, this study aims to explore its mechanism.

**Methods:**

C57BL/6 mice were given intraperitoneal injection of recombinant human EPO at 10000 IU/kg/day, and the control mice were treated with normal saline (vehicle). After 3 weeks, the spleens were harvested. Pathological changes in histology were observed using Hematoxylin and Eosin (H&E) staining. The differential expression genes (DEGs) were identified using RNA sequencing (RNA-Seq), verified with the real-time quantitative polymerase chain reaction (RT-qPCR). The functional-enrichment analysis including Gene Ontology (GO), Kyoto Encyclopedia of Genes and Genomes (KEGG), and Reactome enrichment analysis were performed to reveal the functional characteristics and related biological pathways of DEGs. Immunohistofluorescence (IHF) and flow cytometry (FCM) were used to detect immune cell subsets and proliferation markers.

**Results:**

EPO treatment resulted in splenomegaly, spleen microstructure disorder, splenic corpuscular atrophy, indistinct germinal center, and unclear boundary between white and red pulp structures. RNA-Seq showed that EPO treatment suppressed gene expression associated with immune responses, while promoted cell cycle and DNA replication. IHF and FCM validated that, at the cellular level, T, B, M1 cells were significantly reduced, and M2 cells were significantly decreased after EPO treatment. The proliferation analysis showed that the portion of EDU^+^ or Ki-67^+^cells consisted of granulocytes and macrophages, and after EPO treatment, only macrophages showed a significant increase in their number and proportion, while granulocytes did not show a significant response to EPO stimulation.

**Conclusion:**

Long term high-dose EPO treatment may lead to splenomegaly and immunosuppression of the local immune microenvironment in mice. The mechanism may be related to the increased anti-inflammatory and immunomodulatory functions caused by M2 cells. The study provides, for the first time, the transcriptomic characteristics and immunological of the spleens of EPO treated mice, providing a new perspective for the study of the effects of EPO on mice.

## Introduction

Erythropoietin (EPO) is a glycoprotein hormone produced primarily by the kidneys to promote the production of red blood cells (RBCs), which stimulates the production of RBCs by binding to surface receptors of erythroid progenitor cells in the bone marrow and maintains hemoglobin concentrations within normal ranges (1). In clinical practice, recombinant human erythropoietin (rHuEPO) is used to treat anemia associated with chronic kidney disease, chemotherapy-induced anemia in cancer patients, and anemia in patients with human immunodeficiency virus/acquired immune deficiency syndrome (HIV/AIDS) (2–4). It is also used to reduce the need for blood transfusions in surgical patients and to stimulate bone marrow function after transplantation (5). However, EPO may also cause adverse reactions such as increased blood pressure (6). Based on these, EPO had been used to study its effects on different diseases and to construct different animal models (7, 8). For example, in the rat model of lumbar disc herniation, EPO had the protective effect on neural function (9), and in the mouse model of hypoxia, its effect on hypoxia-induced erythropoietic response was evaluated (10). Recently, a new model of abdominal aortic aneurysm (AAA) has been developed by using EPO injection (11–13). When using this model, we found that EPO treated mice showed significant splenomegaly. This is an interesting phenomenon, and its mechanism has not been reported. In fact, the spleen plays a crucial role in immune response, filtration of blood, and storage of platelets (14). Splenomegaly, the enlargement of the spleen, is a common pathological finding that can arise from a variety of underlying causes, including infectious, hematological, immunological, and neoplastic disorders (15). EPO has been shown to exert pleiotropic effects beyond red blood cell production, including modulation of immune responses and cellular proliferation (16, 17). In the context of EPO-induced splenomegaly in murine models, the enlargement of the spleen may be attributed to several interrelated processes, including increased splenic function (e.g., extramedullary hematopoiesis), abnormal blood flow (e.g., portal hypertension), accumulation of abnormal proteins or lipids, and infiltration of benign or malignant cells (18–20).

In this study, to elucidate the underlying mechanisms, we employed transcriptomic analysis, which revealed a strong association between EPO-induced splenomegaly and immunosuppression of the spleen. This finding suggests that EPO not only drives erythropoietic expansion but also modulates immune function within the splenic microenvironment. Through subsequent immunological validation, we confirmed that EPO treatment leads to a marked alteration in the composition and activity of immune cells within the spleen. Specifically, we observed a downregulation of key immune response pathways and a reduction in the population of effector immune cells, indicating a state of immunosuppression.

## Methods

### Experimental animals and EPO injection

All animal studies were ethically approved by the Animal Care Ethics Committee of Bengbu Medical University. The Animal Ethical Approval number was 2020-050. A total of 63 healthy male C57BL/6 mice (10-week-old, weighing 30-32g) were provided from Chang Zhou Cavens Laboratory Animal Ltd. The animals are housed in ventilated cages with 12-hour light and dark cycles and free access to food and water, four mice in every cage. Ambient room temperature was maintained at 20 to 22°C and humidity at 30 to 70%. The mice were randomly divided into 2 groups using a random number table: vehicle group (Veh, n = 28) was given normal saline; and EPO group (EPO, n = 35) was given intraperitoneal injection of recombinant human EPO (3SBio Inc., Shenyang, China) at 10000 IU/kg/day for 4 weeks (13).

### Specimen collection and preparation

Three weeks post-treatment, the mice were euthanized with a cocktail of ketamine (80 mg/kg)/xylazine (10 mg/kg) intraperitoneally injection and perfused with 10 ml cold 0.01 M phosphate buffer saline (PBS, pH 7.4). For RNA sequencing (RNA-Seq) and/or real-time quantitative polymerase chain reaction (RT-qPCR), the spleens were removed for immediate use or -80°C freezing. For flow cytometry (FCM), the samples were put into the 45-μm nylon mesh and fully ground with the syringe plunger to obtain single cell suspensions. The Percoll (Solarbio, Beijing China) gradient centrifugation was used to separate the single cells (21, 22). For histological analyses, after PBS perfusion, followed by perfusion with 5 ml ice-cold 4% paraformaldehyde (PFA) in 0.01 M PBS (pH 7.4). Then, the spleens were removed, postfixed 12h in 4% PFA, and transferred to 30% sucrose in 0.01 M PBS (pH 7.4) at 4 °C for 24h. Then, the samples were placed in optimal cutting temperature compound embedding medium (Tissue-Tek, Miles, Elkart, IN) and 6 μm frozen sections were obtained longitudinally using a cryostat (Leica CM1900, Bannockburn, IL), followed by thaw-mounting on poly-L-lysine-coated slides. For 5-ethynyl-2’-deoxyuridine (EdU) labeling, the mice were injected intraperitoneally with 50mg/kg EdU prepared with PBS. After 4 h, the spleens were collected and prepared for FCM and histological analyses as described above.

### Hematoxylin and eosin staining

H&E Staining Kit (Beyotime Biotechnology, Shanghai, China) were used to stain the spleens to observe their morphology. Briefly, the 4% PFA fixed frozen sections of spleens were washed with distilled water for 2 minutes, and stained with Hematoxylin Staining Solution for 7 minutes. Next, the sections were rinsed with tap water to remove the extra staining solution, and immersed in tap water for 10 minutes. After washing with distilled water once for a few seconds, the samples were stained with Eosin Staining Solution for 30 seconds, and washed with 70% ethanol twice. Then, the 70%, 80%, 90%, and absolute ethanol were used for 10 seconds, respectively; and xylene was used for 5 minutes for twice. Finally, the neutral gum was used to mount the slide, and samples were examined under a light microscopy (Jiangnan Novel Optics Co., Ltd. Nanjing, China).

### Ribonucleic acid extraction

Total RNA was extracted from spleen tissue of mice using Trizol reagent (Invitrogen, New Jersey, NJ) according the manufacturer’s instructions. RNA quality was determined using 5300 Bioanalyser (Agilent Technologies, Santa Clara, CA) and quantified using the ND-2000 Spectrophotometer (NanoDrop Technologies, Wilmington, DE). Only high-quality RNA sample (OD260/280 = 1.8~2.2, OD260/230 ≥ 2.0, RQN ≥ 6.5, 28S:18S ≥ 1.0, quantity > 1g) was used to construct sequencing library.

### Library preparation and sequencing

RNA purification, reverse transcription, library construction and sequencing were performed at Shanghai Majorbio Bio-pharm Biotechnology Co., Ltd. (Shanghai, China) according to the manufacturer’s instructions. The RNA-seq transcriptome library was prepared following Illumina^®^ Stranded mRNA Prep, Ligation (SanDiego, CA) using 1μg of total RNA. Shortly, messenger RNA was isolated according to polyA selection method by oligo(dT) beads and then fragmented by fragmentation buffer firstly. Secondly double-stranded cDNA was synthesized using a SuperScript double-stranded cDNA synthesis kit (Invitrogen, CA) with random hexamer primers. Then the synthesized cDNA was subjected to end-repair, phosphorylation and adapter addition according to library construction protocol. Libraries were size selected for cDNA target fragments of 300bp on 2% Low Range Ultra Agarose followed by PCR amplified using Phusion DNA polymerase (New England Biolabs, Ipswich, MA) for 15 PCR cycles. After Quantified by Qubit 4.0, the sequencing library was performed on NovaSeq X Plus platform (PE150) using NovaSeq Reagent Kit (Illumina, San Diego, CA).

### Differential expression analysis and functional enrichment

To identify differential expression genes (DEGs) between two different samples, the expression level of each transcript was calculated according to the transcripts per million reads (TPM) method. RNA-Seq by Expectation-Maximization (RSEM) was used to quantify gene abundances. Essentially, differential expression analysis was performed using the DESeq2. DEGs with |log2FC| ≥ 1 and false discovery rate (FDR) < 0.05 were considered to significantly different expressed genes. In addition, functional-enrichment analysis including Gene Ontology (GO), Kyoto Encyclopedia of Genes and Genomes (KEGG), and Reactome enrichment analysis were performed to reveal the functional characteristics and related biological pathways of DEGs. GO functional enrichment, KEGG pathway analysis, and Reactome enrichment analysis were carried out by Goatools, Python scipy, and ReactomePA, respectively.

### RT-qPCR

The total RNAs of spleens in Veh and EPO groups were extracted as mentioned above. The BeyoRT™ II First Strand cDNA Synthesis Kit with gDNA Eraser (Beyotime Biotechnology) was used for the synthesis of first-strand cDNA except RNA. The BeyoFast™ SYBR Green qPCR Mix (Beyotime Biotechnology) and the ABI 7500 Real-Time PCR System (Applied Biosystems, Waltham, MA, USA) were used to perform RT-qPCR. The PCR primers were shown in **Table 1**. The relative expression of target mRNAs was calculated using ^ΔΔ^Ct method (23).

**Table 1:** Primers used in this study for mRNAs.

Gene	Forward primer 5′ - 3′	Reverse primer 5′ - 3′
*Cd3d*	AGACGCCCAAAGCCAGGAG	AGACGCCCAAAGCCAGGAG
*Cd19*	AGATGAGGAGCTGGCCCAACC	ACTGGGACCCAAGCGAGGATG
*Gzmb*	GTGTGCTATGTGGCTGGTTGG	ACTCCCGATCCTTCTGTACTGTC
*Cd74*	TCTAACCATGAACAGTTGCCCATAC	CAGAGCCACCAGGACAGAGAC
*Ly6g*	CACAGAAGCAAAGTCAAGAGCAATC	AGCATTACCAGTGATCTCAGTATTGTC
*Adgre1*	TTGGCAAGCATCATGGCATACC	GACGGTTGAGCAGACAGTGAATG
*Ccr7*	AGCCAGGACCACCCCATTGTAG	AGCCAGGACCACCCCATTGTAG
*Il1b*	CACTACAGGCTCCGAGATGAACAAC	TGTCGTTGCTTGGTTCTCCTTGTAC
*Tgfb*	ACTGGAGTTGTACGGCAGTG	GGGGCTGATCCCGTTGATTT
*Arg1*	CATATCTGCCAAAGACATCGTG	GACATCAAAGCTCAGGTGAATC
*Birc5*	ACTACCGCATCGCCACCTTC	AAGCCAGCCTCCGCCATTC
*Mki67*	GCTTGGTAATGGACTCAGTTATTGTTG	GTCTTCTTCTTGCCTGTGTCTCTG
*Gapdh*	AATGTGTCCGTCGTGGATCTGA	AGTGTAGCCCAAGATGCCCTTC

### Immunohistofluorescence

The IHF assay was performed as previously described (21, 22). Briefly, the 4% PFA fixed frozen sections of spleens were incubated with primary antibodies (**Table 2**) overnight at 4°C. Next day, after washing two times using PBS, incubated with rhodamine (RHO)-conjugated secondary antibodies (**Table 1**) at room temperature for 1 hour. After washing two times using PBS, the sections were coverslipped with ProLong™ Gold Antifade Mountant with DAPI (Thermo Fisher Scientific, Waltham, MA), and examined using a ZWISS Axio observation microscope (Carl Zeiss, Oberkochen, Germany).

**Table 2:** Antibodies used in this study.

Antigen	Host Species and Clone	Cat. # or Lot#	RRID	Conjugation	Source	Used concentration	Methods
CD3	rat monoclonal	12-0032-82	AB_2811741	PE	Invitrogen	0.25 μg/test	FCM
CD3	rat monoclonal	17-0032-82	AB_10597589	APC		0.125 μg/test	
CD11b	rat monoclonal	12-0112-82	AB_2734869	PE		0.125 μg/test	
F4/80	rat monoclonal	11-4801-82	AB_2637191	FITC		0.5 μg/test	
F4/80	rat monoclonal	17-4801-82	AB_2784648	APC		0.25 μg/test	
CD4	rat monoclonal	11-0041-82	AB_464892	FITC		0.25 μg/test	
CD45	rat monoclonal	A15395	AB_2534409	APC-Cyanine7		0.125 μg/test	
B220	rat monoclonal	17-0452-82	AB_469395	APC		0.125 μg/test	
Ly-6G	rat monoclonal	17-9668-82	AB_2573307	APC		0.125 μg/test	
CD68	rat monoclonal	MA5-16676	AB_2538170	FITC		0.25 μg/test	
CCR7	rat monoclonal	47-1971-82	AB_2573974	APC-eFluor™ 780		0.25 μg/test	
CD163	rat monoclonal	17-1631-82	AB_2784646	APC		0.125 μg/test	
CD68	rat monoclonal	12-0681-82	AB_2572569	PE		0.25 μg/test	
Ki-67	rat monoclonal	11-5698-82	AB_11151330	FITC		0.25 μg/test	
IgG2a kappa Isotype Control	rat	17-4321-81	AB_470181	APC		0.125 μg/test	
IgG2a kappa	rat	47-4321-82	AB_1271997	APC-eFluor™ 780		0.25 μg/test	
IgG2b kappa Isotype Control	rat	12-4031-82	AB_470042	PE		0.25 μg/test	
IgG2b kappa Isotype Control	rat	11-4321-80	AB_1834375	FITC		0.25 μg/test	
CD3	rabbit monoclonal	MA5-44535	AB_2926665		Invitrogen	1:200	IHF
CD19	rabbit polyclonal	PA5-27442	AB_2544918				
Ly-6G	rabbit polyclonal	PA5-141170	AB_2932621				
F4/80	rabbit monoclonal	MA5-16363	AB_2537882				
CD68	rat Monoclonal	14-0681-82	AB_2572857				
Arg1	rabbit polyclonal	PA5-29645	AB_2547120				
CCR7	rabbit polyclonal	Ab32527	AB_726208				
Ki-67	rat monoclonal	11-5698-82	AB_11151330	FITC			
Rat IgG (H+L)	goat polyclonal	31629	AB_228240	FITC			
Rabbit IgG (H+L)	donkey polyclonal	A-21207	AB_141637	Alexa Fluor™ 594			

### FCM

The spleen cell suspension obtained in section “specimen collection and preparation” was used for further identify different immune cells using FCM. **Table 2** showed the fluorescent labeled antibodies and the isotype control immunoglobulins used in this study. Briefly, the panel of isotype control immunoglobulins and fluorescent-labeled antibodies were added to the 200 µL of the cell suspension according to the dilution ratios, and incubated at room temperature, protected from light, for 30 minutes. Then, the cells were washed twice with 5 ml PBS for 5 minutes at 400g. Finally, the cells were fixed with 2% PFA. The cells were collected using a flow cytometer (RaiseCare Biotechnology Co., Ltd, Qingdao, China). The data were analyzed using Raiseflower software (RaiseCare Biotechnology Co., Ltd).

### EdU proliferation assays

For the spleen cell suspension derived from EDU-injected mice in section “specimen collection and preparation”, the cells were first stained with corresponding fluorescent labeled antibodies (**Table 2**) as above (FCM), fixed with 4% PFA, and prepared to EDU detection. For EdU detection, the click reaction solution was prepared according to the instructions. The ingredients need to add in the order (for example, a total volume of 1ml requires Click Reaction Buffer 838 μl, CuSO4 40 μl, Azide 488 2 μl, and Click Additive Solution 100 μl, respectively). The click reaction solution (200 μl/tube) was used to label EdU-positive cells by incubating for 30 minutes at room temperature. After washing with 5 ml PBS for two times, the cell markers and EDU could be detected using FCM as above.

For frozen samples derived from EDU-injected mice in section “specimen collection and preparation”, the sections were first with 4% PFA at room temperature for 15 minutes, and prepared to EDU detection. For EdU detection, the click reaction solution was prepared same as above. After removing the fixative solution and wash thrice with PBS for 3 minutes each, the sections were added an appropriate amount of permeabilization solution (PBS containing 0.3% Triton X-100, 200 μl/section), and incubate at room temperature for 15 minutes. Then, the permeabilization solution was removed and washed 2 times with PBS for 3 minutes each, and detected the EDU with the click reaction solution as described in FCM above. After washing with PBS for 3 times (5 minutes each), the sections were coverslipped and examined as described in IHF above.

## Results

### EPO injection resulted in splenomegaly in mice

**Figure 1:**
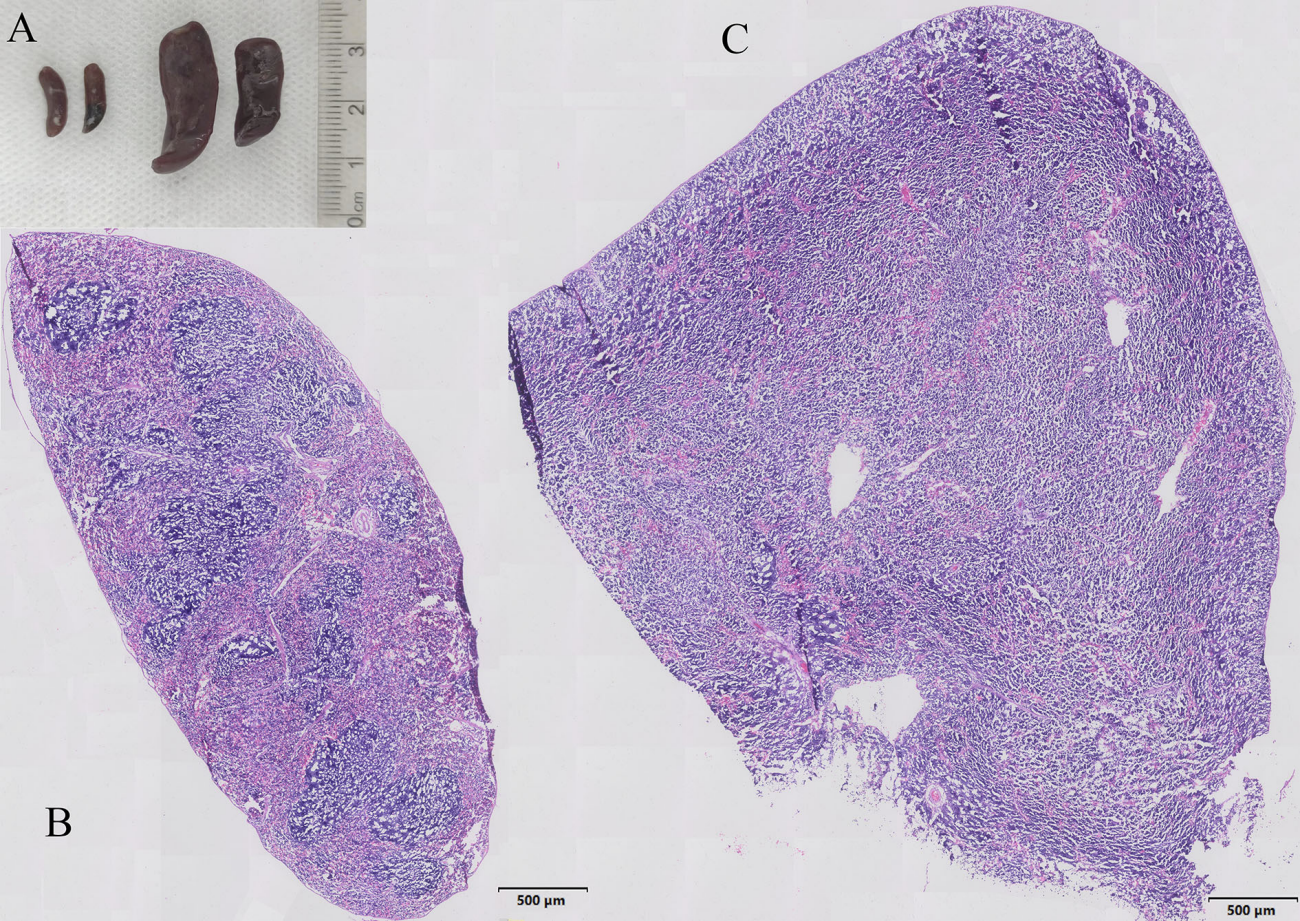
Characteristics of EPO induced splenomegaly in mice. **(A)** Representative photographs of spleen specimens from Veh and EPO groups. **(B, C)** Representative H&E staining pictures of the spleen in Veh **(B)** and EPO **(C)** groups.

In EPO group, all mice were found splenomegaly, while no splenomegaly was found in the Veh group. Representative photographs of spleen specimens from two groups were shown in the **Figure 1A**. H&E staining showed that the spleen structure of the Veh group was clear, the white pulp and red pulp were evenly distributed, the lymphatic follicles were obvious, and the structure of the marginal area and the splenic cord were clearly distinguished-looking (**Figure 1B**). Comparing with Veh group, EPO treatment resulted in spleen microstructure disorder, splenic corpuscular atrophy, indistinct germinal center, and unclear boundary between white and red pulp structures (**Figure 1C**). These results suggest that EPO can affect and change the histological structure of spleen.

### Effect of EPO treatment on gene expression of the spleen

**Figure 2:**
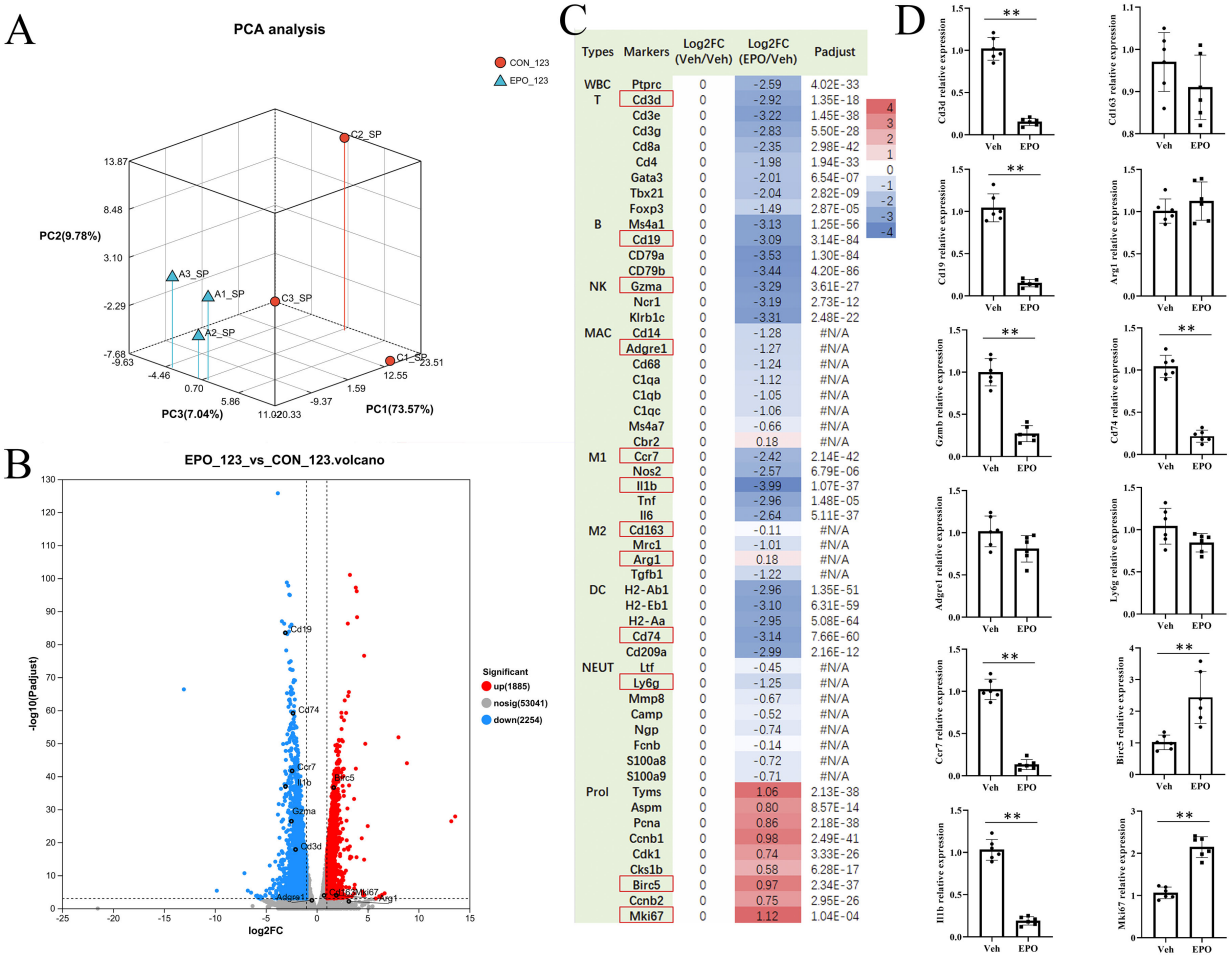
EPO treatment changes the expression of genes in the spleen. **(A)** PCA analysis. CON_123: Veh group; EPO_123: EPO group. **(B)** DEGs between Veh and EPO groups. The smaller the *p* value, the larger the −log10 (adjusted *p* value), and the more significant the difference. The gray dots represent genes with no significant difference, the red dots represent significantly up-regulated genes, and the blue dots represent significantly down-regulated genes. **(C)** The numbers in the figure represent the log2 ratio of various gene expression levels. According to the log2 ratio of gene expression levels relative to the Veh group, the log2 ratio of all genes in the Veh group is “0”, and their color is white; Red indicates high gene expression, while blue indicates low gene expression. *p*adjust < 0.001 was considered statistically significant. The differential analysis software was DESeq2. #N/A: no statistical difference. **(D)** Verify the representative DEGs (red box) in **(A)** using RT-qPCR. The relative expression levels of the target gene to the reference gene Gapdh were calculated using the 2^- ΔΔ^ Ct method. Data represent the mean ± SD (n = 6). **P < 0.01 (Student’s t-test).

RNA-Seq was performed to explore the possible mechanism of splenomegaly induced by EPO. All sequencing data underwent quality control measures and standardization prior to analysis. Principal component analysis (PCA) showed that the samples in each group were clustered and the groups could be clearly separated (**Figure 2A**), indicating that the samples in each group were reproducible. In addition, the transcriptome correlation coefficients between the control group (C1, C2, C3) and the EPO treatment group (A1, A2, A3) were all higher than 0.97 (**Supplementary Figure S1A**), indicating that the sample repeatability met the expectation. Subsequently, with the correction *P* value < 0.05 and Fold change ≥ 2 as the screening criteria, 1885 genes and 2254 genes in EPO group were significantly up-regulated and down-regulated compared with the control group (**Figure 2B**; **Supplementary Table S1**). The cluster analysis heatmaps showed significant differences in mRNA between the EPO and control groups, as shown in **Supplementary Figure S1B**, with heatmap colors representing standardized gene expression from high (red) to low (blue). In order to verify the RNA-Seq results, the DEGs of the main immune cell population and proliferation related markers in the spleen were selected (**Figure 2C**), and 12 representative markers, namely *Cd3d* (T), *Cd19* (B), *Gzma* (NK), *Adgre1* (macrophages, MAC), *Ccr7* and *Il1b* (M1 macrophages, M1), *Cd163* and *Arg1* (M2 macrophages, M2), *Cd74* (dendritic cells, DC), *Ly6G* (Neutrophils, NEUT), and *Birc5* and *Mki67* (proliferation, Prol) were chosen for RT-qPCR identification. **Figure 2C** showed that EPO treatment downregulated the marker gene expressions of total white blood cells (WBC), T, B, NK, M1, and DC, and upregulated the gene expressions related to proliferation (all *P*adjust < 0.05), while had no significant effect on the expression of genes related to MAC, M1, and NEUT (all *P*adjust > 0.05). The RT-qPCR validation results indicated that the expression pattern of the selected representative genes was consistent with the RNA-Seq (**Figure 2D**). This provides preliminary evidence that the results of RNA-Seq are reliable.

### Enrichment analysis of DEGs

To further investigate the mechanism of EPO induced splenomegalysis in mice, GO, KEGG, and Reactome enrichment analysis were performed.

**Figure 3:**
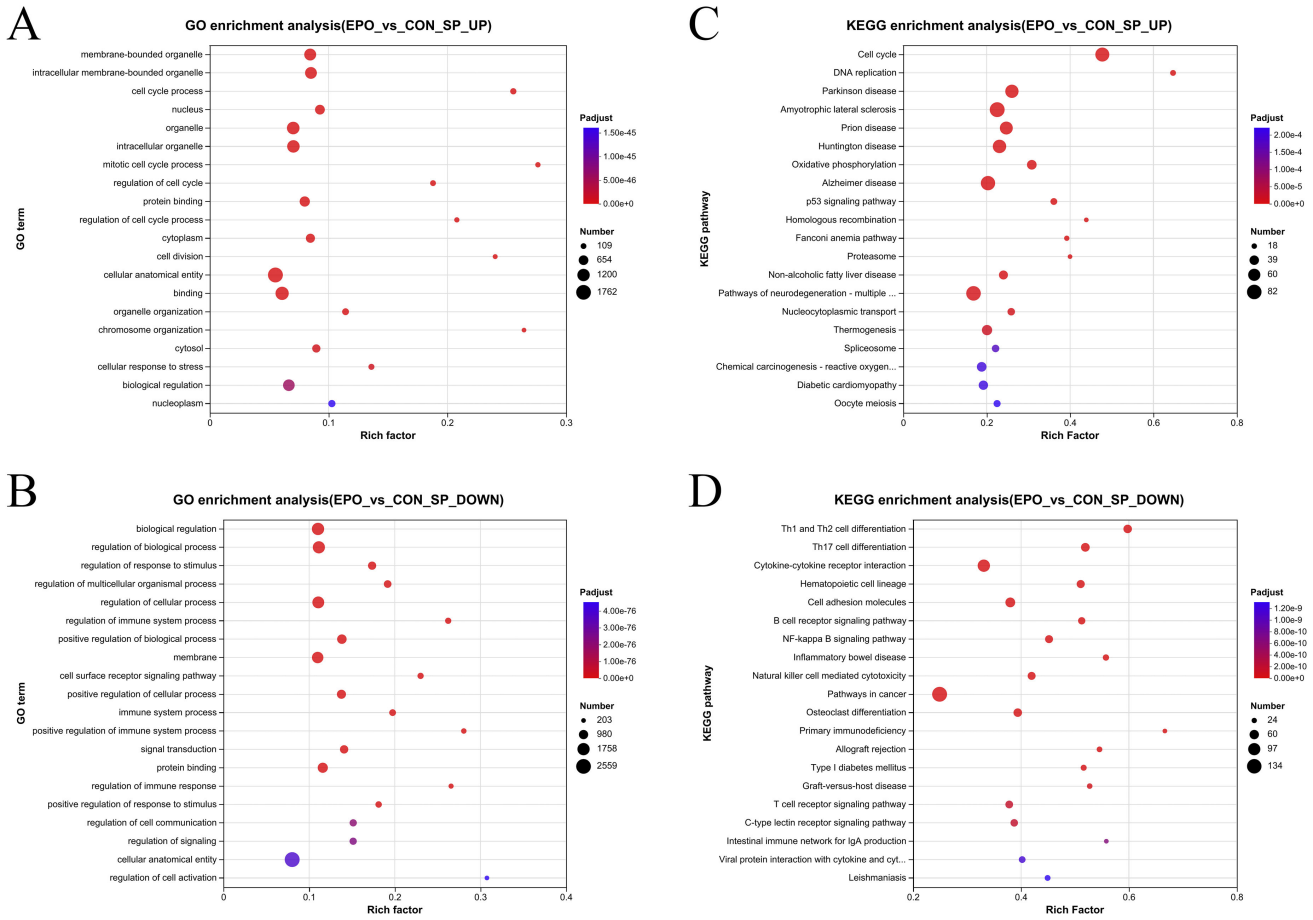
Gene ontology enrichment analysis and KEGG enrichment analysis of DEGs. **(A, B)** Bubble plot of top 20 GO terms in up-regulated **(A)** and down-regulated **(B)** DEGs. **(C, D)** Bubble plot of top 20 KEGG terms in up-regulated **(C)** and down-regulated **(D)** DEGs. The vertical axis represents the GO term or KEGG term and the horizontal axis represents the Rich factor. The size of the dots indicates the number of genes.

Go enrichment analysis showed that compared with the control group, EPO treated mice had significantly increased pathways related to cell cycle and proliferation. For example, membrane-bounded organelle, intracellular membrane-bounded organelle, cell cycle process (**Figure 3A**; **Supplementary Table S2**), However, signaling pathways such as regulation of immune system process, immune system process, regulation of immune response, etc. related to immune function were reduced (**Figure 3B**; **Supplementary Table S3**).

KEGG enrichment analysis showed that the up-regulated DEGs are most enriched in Cell cycle, DNA replication, Parkinson disease, Amyotrophic lateral sclerosis, Oxidative phosphorylation and other pathways (**Figure 3C**; **Supplementary Table S4**); while the down-regulated DEGs were most enriched in adaptive immune-related pathways, such as Th1 and Th2 cell differentiation, Th17 cell differentiation, Cytokine-cytokine receptor interaction, and B cell receptor signaling pathway (**Figure 3D**; **Supplementary Table S5**).

Similarly, Reactome functional annotation analysis found that compared with the control group, upregulated DEGs in the EPO group were most enriched in cell cycle-related pathways, such as M Phase, S Phase, etc. (**Supplementary Figure S2A**; **Supplementary Table S6**). Down-regulated DEGs are most enriched in Immune System, Signal Transduction, Immunoregulatory interactions between a Lymphoid and a non-Lymphoid cell, etc. (**Supplementary Figure S2B**, **Supplementary Table S7**).

Combining the above analysis results, it can be inferred that that EPO treatment suppresses cells associated with immune responses, while promotes cell cycle and DNA replication.

### Effect of EPO treatment on the immune cell populations in mice spleen

**Figure 4:**
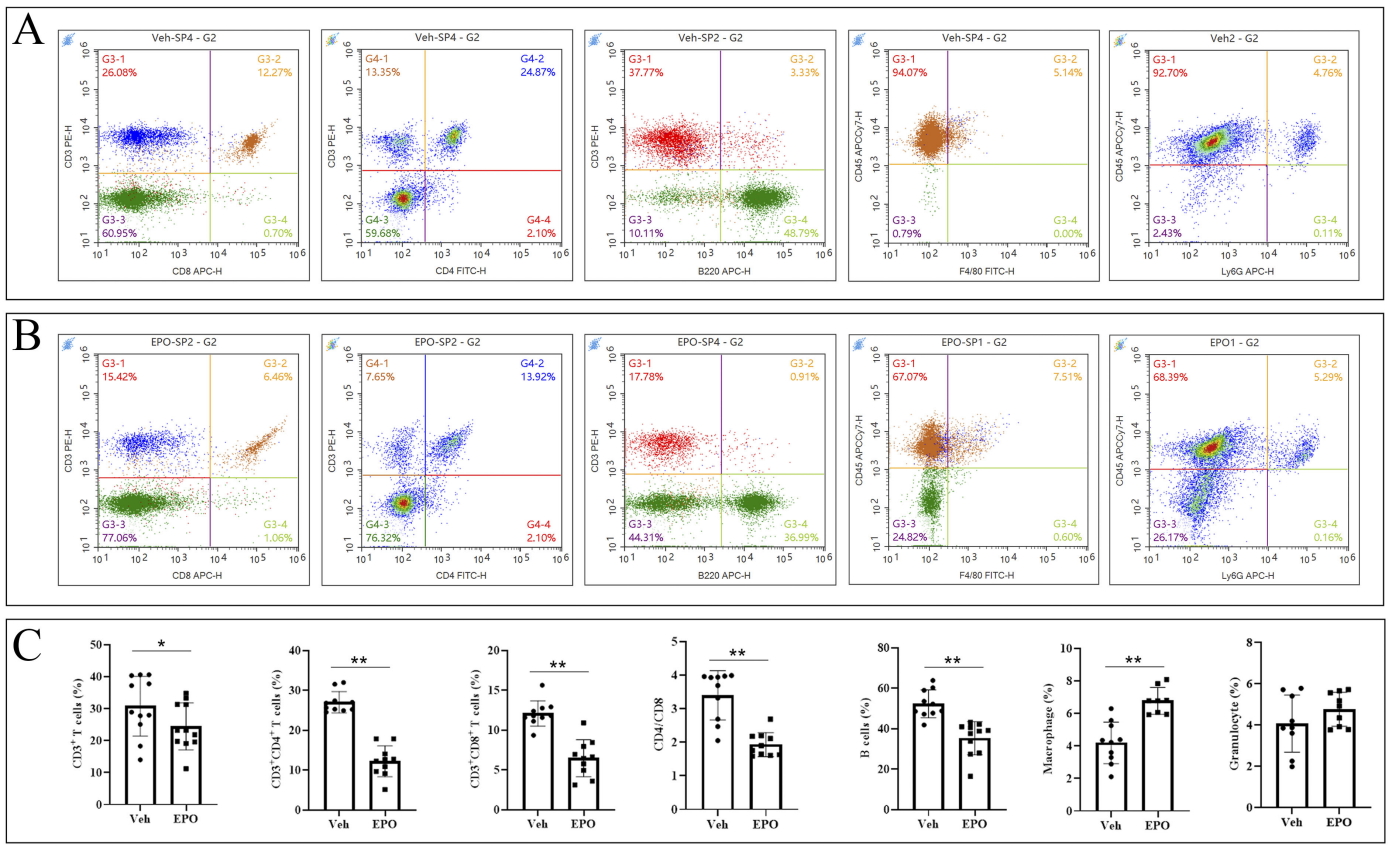
Effect of EPO on mouse spleen immune cells: FCM analysis. **(A, B)** Representative images of FCM in Veh **(A)** and EPO **(B)** groups. **(C)** Quantitative analysis of the indicated cells. Data represent the mean ± SD (n = 6). *P < 0.05, **P < 0.01 (Student’s t-test).

To validate the effect of EPO treatment on mouse spleen immune cell subpopulations at the cellular level, a set of cell markers were detected using FCM. As shown in **Figure 4A** (Veh group) and B (EPO group), CD3^+^ (total T cells), CD3^+^CD8^+^ (cytotoxic T cells, Tc), CD3^+^CD4^+^ (helper T cells, Th), CD3^-^B220^+^ (B cells), CD45^+^F4/80^+^ (macrophages), CD45^+^Ly-6G^+^ (granulocytes) were identified, respectively. The statistical results (**Figure 4C**) showed that the percentages of total T, Tc, Th, B, macrophages, and granulocytes were 30.79 ± 9.29, 12.10 ± 1.60, 27.05 ± 2.67, 52.29 ± 6.87, 4.20 ± 1.28, and 4.07 ± 1.39 in Veh group, respectively. In EPO group, they were 24.43 ± 7.33, 6.48 ± 2.32, 12.23 ± 3.88, 35.29 ± 8.22, 6.78 ± 0.83, and 4.76 ± 0.82, respectively. The comparison of the results between the two groups showed that the total T, Th, Tc, and B cells were significantly reduced in the EPO group (all *p* < 0.01 or 0.05, n = 8), macrophages were significantly increased in the EPO group (*p* < 0.01, n = 8), while granulocytes had no significant differences (*p* > 0.05, n = 8). In addition, the Th/Tc (CD4/CD8) ratio was also statistically analyzed, and the results showed that the Veh group and EPO group were 3.40 ± 0.73 and 1.93 ± 0.36, respectively, with the EPO group significantly lower than that of Veh group (*p* < 0.01, n = 8).

**Figure 5:**
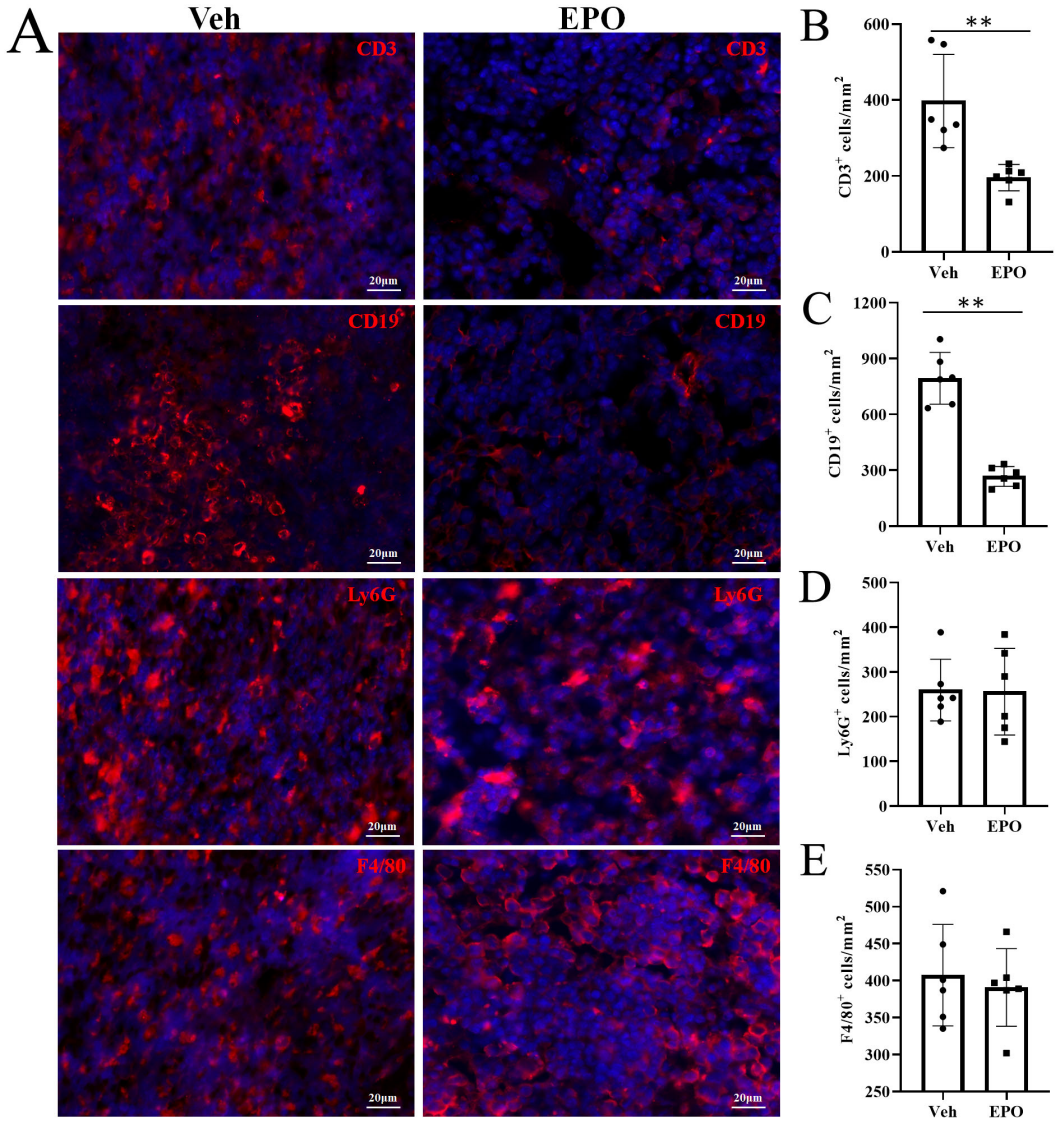
Effect of EPO on mouse spleen immune cells: IHC analysis. **(A)** Representative images of CD3, CD19, Ly-6G, and F4/80 (red) in Veh and EPO groups. **(B–E)** Quantitative analysis of the indicated cells. Data represent the mean ± SD (n = 6). **P < 0.01 (Student’s t-test).

The T, B, granulocytes, and macrophages were further detected by IHF using CD3, CD19, Ly-6G, and F4/80 markers, respectively (**Figure 5A**). The statistical results (**Figures 5B-E**) showed that the numbers (cells/mm^2^) of T, B, granulocytes, and macrophages were 397.33 ± 122.86, 794.00 ± 139.28, 259.50 ± 69.14, and 407.33 ± 68.58 in Veh group, respectively. In EPO group, they were 195.33 ± 34.73, 267.50 ± 53.38, 256.00 ± 97.02, and 390.83 ± 52.48, respectively. The comparison of the results between the two groups showed that the T and B cells were significantly reduced in the EPO group (both *p* < 0.01, n = 6), while granulocytes and macrophages had no significant difference between the two groups (both *p* > 0.05, n = 6).

**Figure 6:**
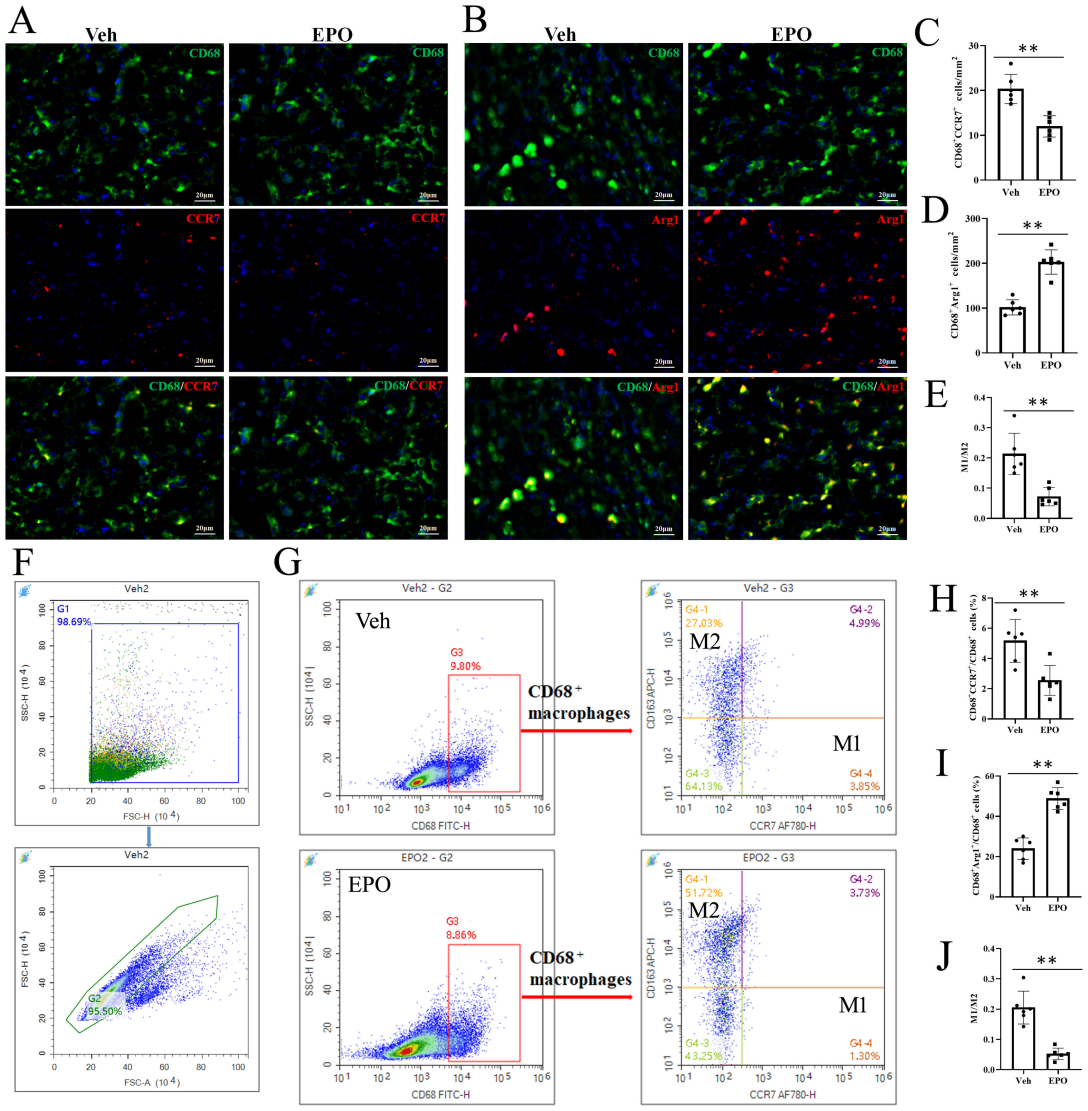
Effect of EPO on mouse spleen on M1 and M2 cells. **(A, B)** Representative images of CD68 (green) and CCR7 (red) **(A)** or Arg1 (red) **(B)** staining in Veh and EPO groups. **(C-E)** Quantitative analysis of CD68^+^CCR7^+^ M1 cells **(C)**, CD68^+^Arg1^+^ M2 cells **(D)**, and M1/M2 ratio **(E)**. **(F, G)** The analysis strategy of FCM. The total spleen cells were gated in G1, the total single spleen cells in G2 **(F)**. Then, CD68^+^ cells were further gated in G3, CD68^+^CCR7^+^ M1 and CD68^+^CD163^+^ M2 cells were further defined **(G)**. **(H-J)** Quantitative analysis of CD68^+^CCR7^+^ M1 cells **(H)**, CD68^+^CD163^+^ M2 cells **(I)**, and M1/M2 ratio **(J)**. Data represent the mean ± SD (n = 6). **P < 0.01 (Student’s t-test).

To further determine the effect of EPO treatment macrophage polarization, CD68 with CCR7 or Arg1 was labeled by IHF. Here, CD68^+^CCR7^+^ cells were M1 cells (**Figure 6A**) and CD68^+^Arg1^+^ cells were M2 cells (**Figure 6B**) (24, 25). The statistical results showed that the numbers of CD68^+^CCR7^+^ M1 cells were 20.33 ± 3.27 and 12.00 ± 2.37 in Veh and EPO groups, respectively (**Figure 6C**); the numbers of CD68^+^Arg^+^ M2 cells were 101.83 ± 16.93 and 203.00 ± 27.20 in Veh and EPO groups, respectively (**Figure 6D**); and the M1/M2 ratios were 0.21 ± 0.07 and 0.07 ± 0.03, respectively (**Figure 6E**). Flow cytometry (FCM) was also used to analyze M1 and M2 cells. **Figures 6F** and G showed the analysis strategy. The total spleen cells were gated in G1, the total single spleen cells in G2 (**Figure 6F**). Then, CD68^+^ cells were further gated in G3, CD68^+^CCR7^+^ M1 and CD68^+^CD163^+^ M2 cells were further defined (the representative images were shown in **Figure 6G**). The statistical results showed that the percentages of CD68^+^CCR7^+^ M1 cells were 5.17 ± 1.42 and 2.55 ± 0.99 in Veh and EPO groups, respectively (**Figure 6H**); the percentages of CD68^+^CD163^+^ M2 cells were 23.97 ± 5.34 and 48.86 ± 5.44 in Veh and EPO groups, respectively (**Figure 6I**); and the M1/M2 ratios were 0.21 ± 0.05 and 0.05 ± 0.02, respectively (**Figure 6J**). EPO treatment significantly decreased the number or percentage of M1 cells, increased M2 cells, and reduced the M1/M2 ratio (all *p* < 0.01, n = 6, vs. Veh).

### Effect of EPO on the proliferation of different immune cells in mouse spleen

**Figure 7:**
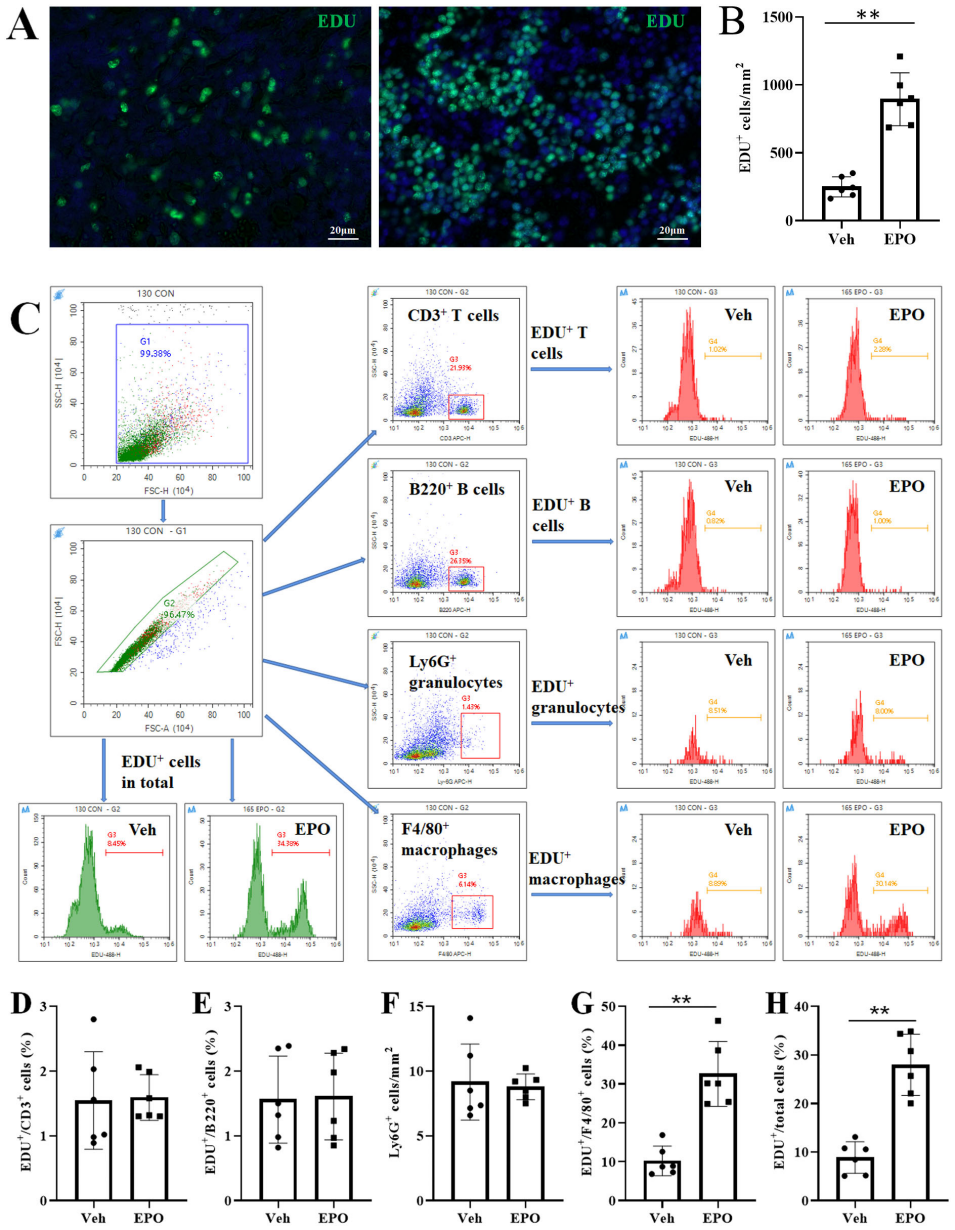
Effect of EPO on the proliferation of different immune cells in mouse spleen: EdU proliferation assays. **(A)** Representative images of EDU-positive cells (green). **(B)** Quantitative analysis of EDU^+^ cells. **(C)** The total spleen cells were gated in G1, the total single spleen cells in G2, CD3^+^ T, B220^+^ B, Ly-6G^+^ granulocytes, and F4/80^+^ macrophages, were in G3, respectively. The representative FCM histograms of EDU^+^ cells in total single spleen cells were shown in the first two images in the third row. The representative histograms of EDU^+^ T, EDU^+^ B, EDU^+^ granulocytes, and EDU^+^ macrophages were shown in the images of the two columns on the right. **(D-H)** Quantitative analysis of the percentages of EDU^+^ cells in CD3^+^ T **(D)**, B220^+^ B **(E)**, Ly-6G ^+^ granulocytes **(F)**, F4/80^+^ macrophages **(G)**, and total single spleen cells **(H)**, respectively. Data represent the mean ± SD (n = 6). **P < 0.01 (Student’s t-test).

To validate the effect of EPO on Cell cycle and DNA replication, the EdU proliferation assays were used. As shown in **Figure 7A**, the EdU-positive (green) cells were relatively sparse in the spleen slices of the Veh group, but significantly increased in the EPO group. The statistical results in **Figure 7B** indicated that there was a significant difference between the two groups (249.50 ± 74.32 vs 894.33 ± 195.07, *p* < 0.01, n = 6). FCM was also used for EDU proliferation analysis, with the aim of verifying the results of slice staining and comparing the proliferation of different immune cell subpopulations. **Figure 7C** showed the analysis strategy. The statistical results (**Figures 7D–F**) showed that the percentages of EDU^+^ cells in CD3^+^ T, B220^+^ B, Ly-6G^+^ granulocytes were 1.55 ± 0.75, 1.56 ± 0.67, and 9.15 ± 2.93, respectively, in the Veh group, while they were 1.54 ± 0.42, 1.61 ± 0.67, and 8.79 ± 0.99, respectively, in the EPO group. There were no statistically significant differences in the positivity rate within the same cell subsets between the two groups (all *p* > 0.05, n = 6). In **Figure 7G**, the percentages of EDU^+^ cells in F4/80^+^ macrophages were 10.21 ± 3.84 and 32.61 ± 8.34 in the Veh group and EPO group, respectively. The EPO group was significantly higher than the Veh group (*p* < 0.01, n = 6). Similarly, the percentages of EDU^+^ cells in in total spleen cells were 8.86 ± 3.24 and 27.97 ± 6.31 in the Veh group and EPO groups, respectively. The EPO group was also significantly higher than the Veh group (**Figure 7H**, p < 0.01, n = 6).

**Figure 8:**
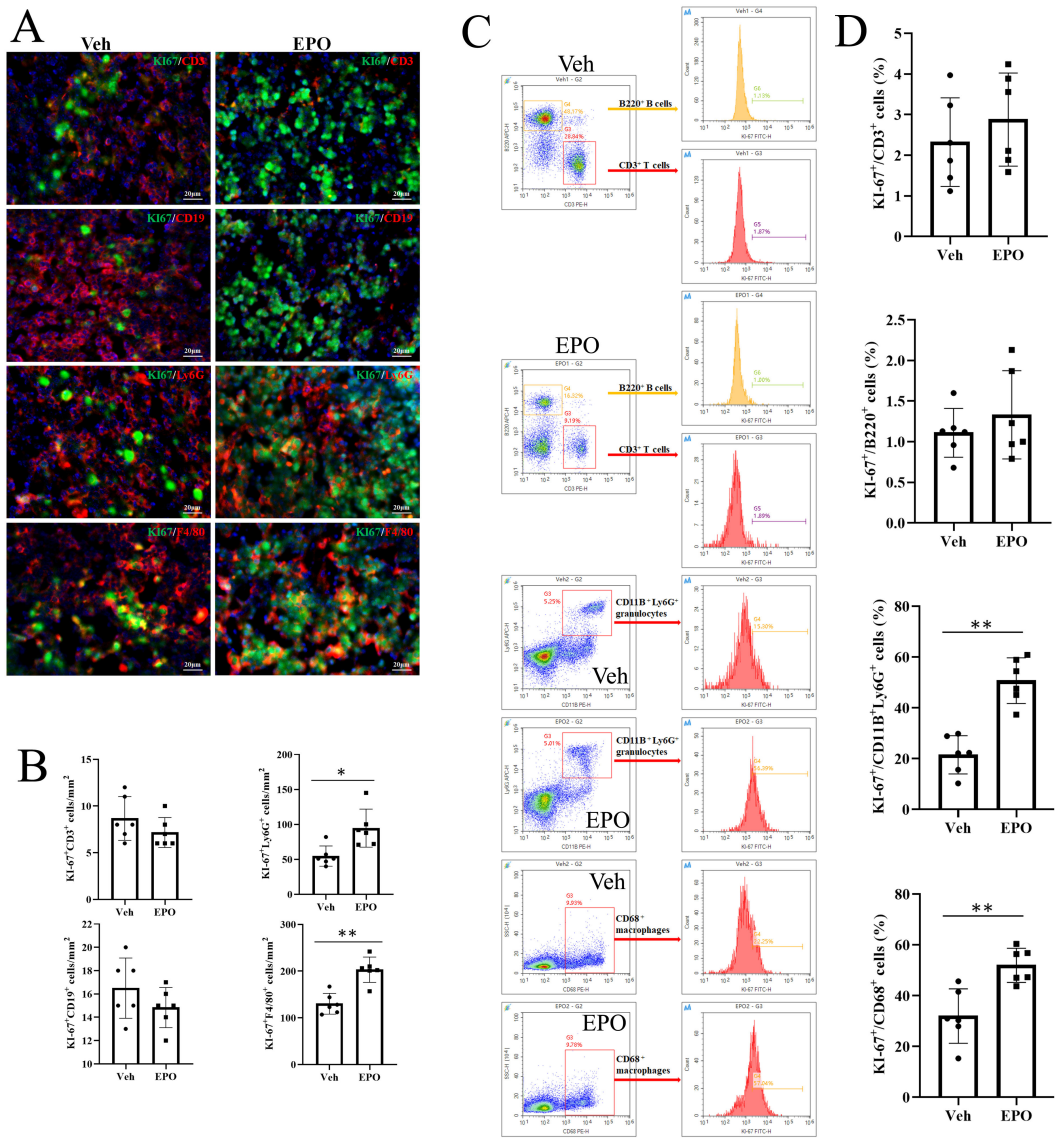
Effect of EPO on the proliferation of different immune cells in mouse spleen: Ki-67 FCM assays. **(A)** Representative images of Ki-67 (green) and CD3 (red), CD19 (red), Ly-6G (red), or F4/80 (red) staining in Veh and EPO groups. **(B)** Quantitative analysis of Ki-67^+^CD3^+^, Ki-67^+^CD19^+^, Ki-67^+^Ly-6G^+^, and Ki-67^+^F4/80^+^ cells. **(C)** The analysis strategy of Ki-67^+^ cell in different immune cell subsets. The gate strategy of total spleen cells (G1) and total single spleen cells (G2) was same as **Figure 7C**, CD3^+^ T, B220^+^ B, CD11B^+^Ly-6G^+^ granulocytes, and CD68^+^ macrophages, were further gated (G3 or G4), respectively. Then, the percentages of Ki-67^+^ cells in T, B, granulocytes, and macrophages were analyzed in histograms. **(D)** Quantitative analysis of the percentages of Ki-67^+^ cells in CD3^+^ T, B220^+^ B, Ly-6G ^+^ granulocytes, and F4/80^+^ macrophages. Data represent the mean ± SD (n = 6). **P < 0.01 (Student’s t-test).

To further determine the results of EdU proliferation assays, Ki-67, a 300 kDa nuclear protein, present during all active phases of the cell cycle (G1, S, G2, and mitosis), but is absent from resting cells (G0), was used by IHF and FCM. **Figure 8A** showed the representative images of the co-expression of different immune cell markers (red) with Ki-67 (green) in the Veh group and EPO groups. The statistical results (**Figure 8B**) showed that the numbers of Ki-67^+^CD3^+^ T and Ki-67^+^B220^+^ B were 8.67 ± 2.34 and 16.50 ± 2.59, respectively, in the Veh group, while they were 7.17 ± 1.60 and 14.83 ± 1.72, respectively, in the EPO group. The numbers of the numbers of Ki-67^+^ T and B cells had no statistically significant differences between the two groups (all *p* > 0.01, n = 6). The numbers of Ki-67^+^Ly-6G^+^ granulocytes and Ki-67^+^F4/80^+^ macrophages were 54.67 ± 14.51 and 130.17 ± 22.23 in the Veh group and EPO group, respectively, in the Veh group, while they were 94.67 ± 27.16 and 203.00 ± 27.20, respectively, in the EPO group. EPO treatment significantly increased the Ki-67^+^ cell numbers in granulocytes and macrophages (both *p* < 0.01, n = 6). **Figure 8C** showed the analysis strategy of Ki-67^+^ cell in different immune cell subsets. The gate strategy of total spleen cells (G1) and total single spleen cells (G2) was same as **Figure 7C**, CD3^+^ T, B220^+^ B, CD11B^+^Ly-6G^+^ granulocytes, and CD68^+^ macrophages, were further gated (G3 or G4), respectively. Then, the percentages of Ki-67^+^ cells in T, B, granulocytes, and macrophages were analyzed in histograms. The statistical results (**Figure 8D**) showed that the percentages of Ki-67^+^ cells in T and B were 2.33 ± 1.09 and 1.11 ± 0.30, respectively, in the Veh group, while they were 2.88 ± 1.15 and 1.33 ± 0.54, respectively, in the EPO group. There were no statistically significant differences in the positivity rate within the same cell subsets between the two groups (both *p* > 0.05, n = 6). The percentages of Ki-67^+^ cells in granulocytes and macrophages were 21.53 ± 7.55 and 31.97 ± 10.73 in the Veh group, respectively, while they were 50.73 ± 8.99 and 51.96 ± 6.74, respectively, in the EPO group. EPO treatment significantly increased the percentages of Ki-67^+^ cells in granulocytes and macrophages (both *p* < 0.01, n = 6).

## Discussion

EPO is an important glycoprotein hormone with multiple biological functions, such as promoting erythropoiesis, antioxidant activity, anti-apoptosis, anti-inflammatory, and promoting angiogenesis (26, 27). Recent studies have shown that high-dose EPO can induce AAA in WT mice, becoming a new animal model for studying AAA (11–13). During the use of this animal model, we found that these mice exhibited significant splenomegaly. The spleen is the largest peripheral immune organ in the body, and its structure and function are closely related to the distribution of immune cells (28, 29). High dose EPO treatment leads to splenomegaly, possibly due to EPO stimulating the proliferation and aggregation of certain immune cells in the spleen, altering its tissue structure and increasing its volume.

In order to investigate the mechanism of EPO induced splenomegaly in mice, RNA sequencing was firstly performed on the splenic tissues of Veh and EPO treatment mice, and the DEGs between the two groups were screened. With corrected P value < 0.05 and Fold change ≥ 2 as screening criteria, compared with the Veh group, 2150 genes were significantly up-regulated and 3226 genes in EPO group down-regulated. Twelve DEGs related to immune response and proliferation were selected for verification, and the results of RT-qPCR indicated that the expression pattern of the selected representative genes was consistent with the RNA-Seq. This provides preliminary evidence that the results of RNA-Seq are reliable.

To further understand the effects of EPO, GO enrichment was used to analyze differentially expressed genes in each group. It can be seen that compared with the Veh group, the EPO treatment upregulated with cell cycle process, mitotic cell cycle process, cell cycle regulation, nucleus and organelles and other signaling pathways related to cell proliferation, while immune-related signaling pathways, such as immune system processes, regulation of immune response, and regulation of immune system processes, were down-regulated. These results indicate that EPO can promote the proliferation of cells, and change the immune function of mouse spleen, so that the immune function of mice is decreased.

Similarly, KEGG enrichment analysis also found that the use of EPO can up-regulate signaling pathways related to Cellular Processes, most of which belong to Cell growth and death pathways, such as Cell cycle, p53 signaling pathway, Oocyte meiosis, etc. Compared with the Veh group, the down-regulated pathways are related to immune function, such as Th1 and Th2 cell differentiation, Th17 cell differentiation, T cell receptor signaling pathway, B cell receptor signaling pathway and other pathways related to immune function.

Similar results were also obtained when Reactome enrichment analysis was used, with significantly up-regulated cell cycle-related signaling and down-regulated immune system-related signaling in the EPO group. It can be seen that the spleen of mice treated with EPO is up-regulated in different links of cell cycle and signaling pathways related to DNA replication, while immune function is down-regulated.

To validate the effect of EPO treatment on mouse spleen immune cell subpopulations at the cellular level, a set of cell markers were detected using IHF and FCM. The results indicated that EPO treated mice showed a decrease in CD4^+^T, CD8^+^T, B, and M1 cells, an increase in M2 cells, and the decreased CD4/CD8 and M1/M2 ratios. Both T and B cells play an important role in adaptive immunity. The classification of T cells is complex and numerous, and they play an important role in immune regulation (30, 31). CD8^+^ T cells, that is, cytotoxic T cells, can kill virus-infected cells or cancer cells by releasing related substances (32, 33). CD4^+^T cells, also known as helper T cells, have many types and play an important role in adaptive immunity (34, 35). B cells produce high-affinity antibodies, act as antigen-presenting cells, and secrete cytokines (36–38). M1 cells play a crucial role in the body’s immune defense, inflammatory response, and anti-tumor effects, while M2 cells play an important role in immune regulation, anti-inflammatory response, and tissue repair (39–42). The CD4/CD8 ratio is an important indicator for evaluating immune system function, and its decrease reflects that the body is in an immunosuppressive state (43, 44). The change in the M1/M2 ratio also reflects the dynamic balance of the body’s immune system. Its decrease can help with anti-inflammatory and tissue repair in some cases, but in other cases it may also indicate that the body is in an immunosuppressive state (45, 46). Based on the above results, it can be concluded that mice treated with EPO are in an immunosuppressive state in the spleen immune microenvironment. This may be related to the different regulation of EPO on the proliferation and differentiation of different immune cell subpopulations.

The analysis results of RNA-Seq precisely indicate that EPO has the effect of promoting cell cycle and proliferation. To validate this phenomenon and clarify the proliferation and differentiation of immune cells, the EdU proliferation assays and Ki-67 staining were used. IHF and FCM further confirmed that the proliferating cells were mainly granulocytes and macrophages. Although granulocytes were in a proliferative state, there was no significant difference between the EPO group and the Veh group, indicating that they did not contribute to the cell proliferation caused by EPO treatment. However, the proliferation of macrophages was significantly higher in the EPO group than in the Veh group, indicating that macrophages are the main contributor to the cell proliferation caused by EPO treatment. This may be related to the expression of EPOR in different immune cell populations, as EPO can activate downstream signaling pathways such as the JAK-STAT pathway by binding to EPO receptors on the cell surface, thereby promoting the expression of cell cycle related genes and pushing cells into the proliferation cycle (47). For example, using EpoR-tdTomato-Cre mice, the study has shown that EPOR can be detected in splenic red pulp macrophages (48). However, EPO has no significant effect on other immune cell types, which may be due to the lower expression of EPOR in these cells, resulting in their lower sensitivity to EPO. Therefore, the proliferation regulatory mechanism of EPO on these cells cannot be activated.

Due to T cells and B cells are the main participants in adaptive immune responses (49), EPO reduces the proportion of T cells and B cells in the spleen, which may affect the body’s adaptive immune function, such as antigen recognition and antibody production. However, further research is needed to clarify the specific degree and mechanism of impact. EPO treatment can increase the proportion of M2 cells in the spleen, but considering that EPO treatment did not increase the total number of macrophages, this suggests that the increase in the proportion of M2 cells may be a relative result of a decrease in the number of other immune cells. However, due to the anti-inflammatory and immunomodulatory functions of M2 macrophages in immune responses (39–42). This not only helps alleviate inflammatory reactions and promote tissue repair, but also leads to immune suppression due to an imbalance in the M1/M2 ratio. The characteristics of EPO in regulating the proportion of immune cells and promoting cell proliferation may provide new ideas for the treatment of certain immune related diseases. For example, in autoimmune diseases, EPO may alleviate excessive immune responses by regulating the balance of immune cells. However, there are still many issues that need further exploration. What is the specific mechanism of action of EPO on different subgroups of immune cells? What is the immunomodulatory effect of EPO in different disease states? The answers to these questions will help to better understand the immunological functions of EPO and provide stronger scientific evidence for clinical applications.

While our findings primarily focus on the local effects of EPO on the spleen, it is important to acknowledge the potential systemic effects of EPO, which may indirectly contribute to the observed splenic changes. EPO is well-known for its primary role in erythropoiesis, stimulating the production of RBCs and increasing hematocrit levels (1). In this study, the high-dose EPO treatment likely led to a significant increase in RBC mass and hematocrit, which may lead to splenic congestion and enlargement, potentially exacerbating the structural disorganization and atrophy of splenic corpuscles. The systemic effects of EPO, including increased hematocrit and potential hypertension, may synergize with its direct effects on the spleen to create a unique immunological microenvironment. For instance, the increased RBC mass and splenic congestion could alter the oxygen tension within the spleen, potentially favoring the polarization of macrophages toward an M2 phenotype (50), which could explain the overall immunosuppressive state observed in the spleens of EPO-treated mice. Additionally, the systemic effects of EPO on blood pressure and vascular function may further modulate the splenic immune response by altering the local cytokine milieu and immune cell trafficking (50, 51). While our study provides novel insights into the local immunological and transcriptomic changes induced by EPO in the spleen, it is important to acknowledge that we did not directly measure systemic parameters such as hematocrit or blood pressure. Future studies should aim to correlate these systemic effects with the observed splenic changes to better understand the interplay between systemic and local responses to EPO.

Our findings of EPO-induced splenomegaly and immunosuppression in mice share both similarities and differences with the splenic pathology observed in polycythemia patients, particularly those with elevated EPO levels. Polycythemia, whether primary (e.g., polycythemia vera) or secondary (e.g., due to chronic hypoxia or EPO-secreting tumors), is often associated with splenomegaly due to increased RBC mass and extramedullary hematopoiesis in the spleen (52). In polycythemia patients, the spleen becomes congested with RBCs, leading to structural disorganization and functional impairment, similar to the microstructural disorder and splenic corpuscular atrophy observed in our EPO-treated mice (53, 54). However, there are notable differences between the murine model and human polycythemia. In polycythemia vera, a primary myeloproliferative disorder, splenomegaly is driven by clonal expansion of hematopoietic stem cells, often independent of EPO levels (55). In contrast, our murine model reflects secondary polycythemia induced by exogenous EPO administration, which directly stimulates erythropoiesis and increases RBC mass. Despite these differences, both conditions result in splenic congestion and structural changes, suggesting that EPO-driven erythropoiesis plays a central role in splenic pathology across species. The immunosuppressive effects observed in our study may parallel immune dysregulation observed in polycythemia patients. Polycythemia patients often exhibit an increased susceptibility to infections and a blunted immune response, which has been attributed to alterations in the immune microenvironment (56). These findings are consistent with our observations in EPO-treated mice, suggesting that EPO-driven immune modulation may contribute to the clinical phenotype of polycythemia patients. Future studies should investigate whether targeting the EPO pathway or modulating the immune microenvironment could ameliorate splenic pathology and immune dysfunction in these patients.

## Conclusion

Our study findings indicate that long term high-dose EPO treatment may lead to splenomegaly and immunosuppression of the local immune microenvironment in mice. The mechanism may be related to the increased anti-inflammatory and immunomodulatory functions caused by M2 cells. The study provides, for the first time, the transcriptomic characteristics and immunological of the spleens of EPO treated mice, providing a new perspective for the study of the effects of EPO on mice.

## Data Availability

The datasets presented in this study can be found in online repositories. The names of the repository/repositories and accession number(s) can be found below: https://www.ncbi.nlm.nih.gov/, sra/PRJNA1194367.

## Supplementary material

Supplementary Figure 1 The transcriptome correlation coefficients and heat map of cluster analysis. **(A)** The transcriptome correlation coefficients. Veh group (C1, C2, C3) and the EPO group (A1, A2, A3). **(B)** Heat map of cluster analysis showed significant differences in mRNA between the two groups. The high-standardized gene expression was shown in red and low-standardized gene expression was blue.

Supplementary Figure 2 Reactome KEGG enrichment analysis of DEGs. **(A, B)** Bubble plot of top 20 Reactome terms in up-regulated **(A)** and down-regulated **(B)** DEGs. The vertical axis represents the Reactome term and the horizontal axis represents the Rich factor. The size of the dots indicates the number of genes.

Supplementary Table 1 The DEGs of two groups.

Supplementary Table 2 GO enrichment analysis of up-regulated DEGs in EPO vs Veh.

Supplementary Table 3 GO enrichment analysis of down-regulated DEGs in EPO vs Veh.

Supplementary Table 4 KEGG enrichment analysis of up-regulated DEGs in EPO vs Veh.

Supplementary Table 5 KEGG enrichment analysis of down-regulated DEGs in EPO vs Veh.

Supplementary Table 6 Reactome enrichment analysis of up-regulated DEGs in EPO vs Veh.

Supplementary Table 7 Reactome enrichment analysis of down-regulated DEGs in EPO vs Veh.
