# Supplementary figures and images for: Immunosuppression of spleen in mice treated with erythropoietin: transcriptomic and immunological analysis
Source: Front Immunol. 2025 Mar 21;16:1560589. doi: 10.3389/fimmu.2025.1560589 (PMC11968373; doi:10.3389/fimmu.2025.1560589)

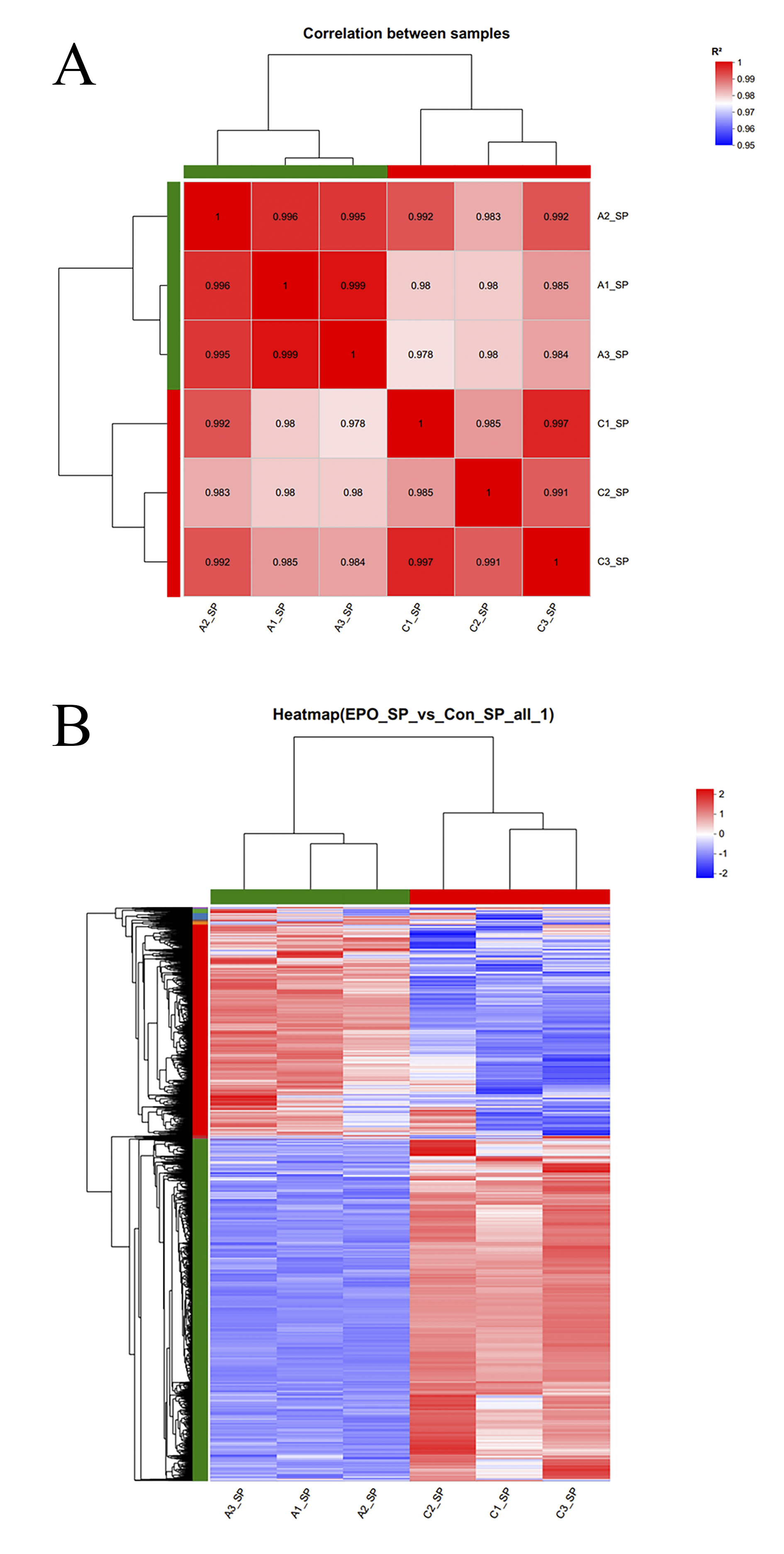

Supplement: Supplementary Figure 1 — The transcriptome correlation coefficients and heat map of cluster analysis. (A) The transcriptome correlation coefficients. Veh group (C1, C2, C3) and the EPO group (A1, A2, A3). (B) Heat map of cluster analysis showed significant differences in mRNA between the two groups. The high-standardized gene expression was shown in red and low-standardized gene expression was blue. [file Image1.tif]

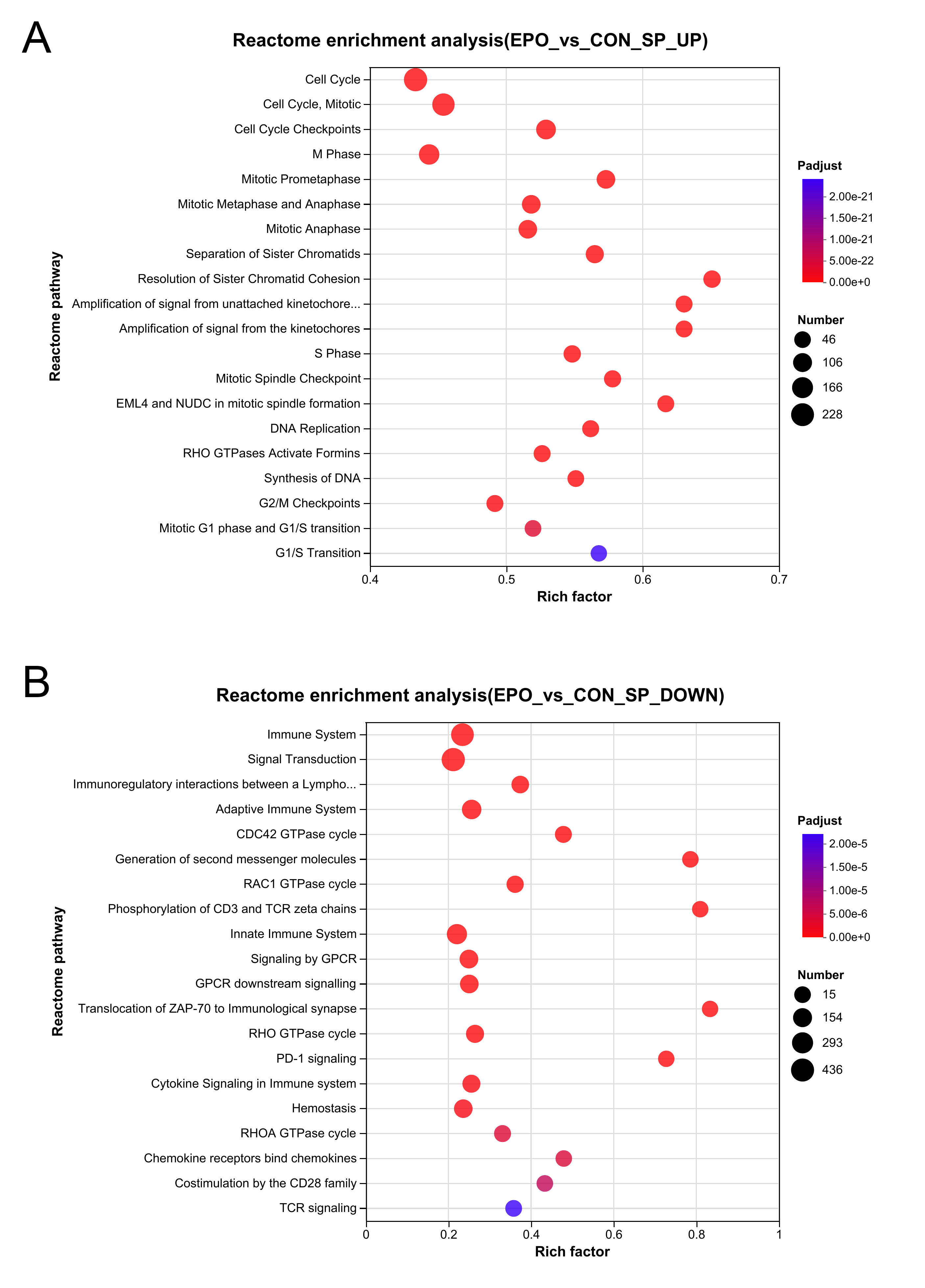

Supplement: Supplementary Figure 2 — Reactome KEGG enrichment analysis of DEGs. (A, B) Bubble plot of top 20 Reactome terms in up-regulated (A) and down-regulated (B) DEGs. The vertical axis represents the Reactome term and the horizontal axis represents the Rich factor. The size of the dots indicates the number of genes. [file Image2.tif]
